# Impaired Function of a Rare Mutation in the *MMUT* Gene Causes Methylmalonic Acidemia in a Chinese Patient

**DOI:** 10.1155/2022/5611697

**Published:** 2022-07-22

**Authors:** Siyu Dai, Yanting Yang, Yaqian Li, Hongqian Liu

**Affiliations:** ^1^Department of Obstetrics and Gynecology, West China Second University Hospital, Sichuan University, Chengdu 610041, China; ^2^Medical Genetics Department/Prenatal Diagnostic Center, West China Second University Hospital, Sichuan University, Chengdu 610041, China; ^3^Key Laboratory of Birth Defects and Related Diseases of Women and Children, Ministry of Education, Sichuan University, Chengdu 610041, China; ^4^Department of Obstetrics/Gynecology, Joint Laboratory of Reproductive Medicine (SCU-CUHK), Key Laboratory of Obstetric, Gynecologic and Pediatric Diseases and Birth Defects of Ministry of Education, West China Second University Hospital, Sichuan University, Chengdu 610041, China

## Abstract

Methylmalonic acidemia (MMA) is an autosomal recessive metabolic disorder mainly caused by mutations in the methylmalonyl coenzyme A mutase (MCM) gene (*MMUT*) and leads to the reduced activity of MCM. In this study, a 3-year-old girl was diagnosed with carnitine deficiency secondary to methylmalonic acidemia by tandem mass spectrometry (MS/MS) and gas chromatography/mass spectrometry (GS/MS). Whole-exome sequencing (WES) was performed on the patient and identified two compound heterozygous mutations in *MMUT*: c.554C>T (p. S185F) and c.729–730insTT (p. D244Lfs^*∗*^39). Bioinformatics analysis predicted that the rare missense mutation of c.554C>T would be damaging. Moreover, this rare mutation resulted in the reduced levels of *MMUT* mRNA and MMUT protein. Collectively, our findings provide a greater understanding of the effects of *MMUT* variants and will facilitate the diagnosis and treatment of patients with MMA.

## 1. Introduction

Methylmalonic acidemia (MMA) is an autosomal recessive metabolic disorder that is mainly caused by inherited defects in methylmalonyl-CoA mutase (MCM, MUT) or metabolic defects in its cofactor adenosylcobalamin [1,2]. Although the clinical manifestations of children with MMA vary, the most common symptoms and signs include recurrent vomiting, lethargy, seizures, failure to thrive, hypotonia, and mental retardation. This disease has a poor prognosis, and all forms of inherited MMA exhibit progressive encephalopathy and secondary hyperammonemia in the early neonatal period. Therefore, an efficient and rapid diagnosis and timely clinical intervention can reduce the mortality associated with MMA.

The most common causes of MMA are mutations in the human *MMUT* gene or mutations in the *MMAA*, *MMAB*, or *MMADHC* genes that lead to an impairment in the transport and synthesis of the MUT cofactor, 5′-deoxyadenosylcobalamin [3]. The *MMUT* gene is located on chromosome 6p12.3, features 13 exons, and encodes MCM protein [4]. Mutations in the *MMUT* gene account for 60–70% of MMA cases [5]. Patient cell lines can be assigned to the *MUT*-type MMA by complementation analysis of fibroblast heterokaryons [6,7]. Patients with *MUT*-type MMA can be divided into two subtypes: *mut*^0^ (with a completely defective MCM protein in fibroblasts) and *mut*^−^ (with residual MCM activity in the presence of high concentrations of cobalamin) [8].

Almost 250 mutations in the *MMUT* gene have been reported in different populations [9]. Previous studies have highlighted that different ethnic groups are associated with different types of mutations in the *MMUT* gene. For example, c.322C>T(p. R108C) is more common in Hispanic patients [10], c.1630–1631delGGinsTA (p. G544X) and c.1280G>A (p. G427D) are more common in Asian patients [10], and c.671–678dup is more common in Spanish patients [11], while c.1863A>T(p. K621N), c.1943G>A(p. G648D), and c.1889G>A(p. G630E) have been frequently detected in Indian patients [1]. Other research studies showed that c.2150G>T(p. G717V) mutation is common in black patients [12]. In China, the most common mutations are c.729–730insTT(p. D244Lfs^*∗*^39), c.1106G>A(p. R369H), c.323G>A(p. R108H), and c.1107dupT(p. T370Yfs^*∗*^2) [13].

This study used whole-exome sequencing (WES) to identify c.554C>T (p. S185 F) and c.729–730insTT (p. D244Lfs^*∗*^39) compound heterozygous mutations in the *MMUT* gene in a patient with MMA. The c.729–730insTT mutation is known to be one of the most common mutations in China [13]. The proband's mother has the heterozygous c.554C>T mutation, and the father carries the heterozygous variation of c.729–730insTT. We further confirmed the diminished expression of MMUT protein and *MMUT* mRNA levels caused by this missense mutation of c.554C>T, thus providing strong evidence for the pathogenicity of this rare missense mutation.

## 2. Materials and Methods

### 2.1. Subjects

First, we acquired signed and informed consent from the proband's family. Then, we acquired peripheral blood samples from the proband and her family members. Our research was approved by the Ethical Review Board of West China Second University Hospital, Sichuan University. A paediatric neuroradiologist evaluated the proband's brain MRI results.

### 2.2. WES and Sanger Sequencing

Genomic DNA was extracted from the peripheral blood samples using a whole-blood DNA purification kit (Axygen Scientific, Union City, San Francisco, CA, USA). WES was performed on the patient and her parents. One microgram of genomic DNA was utilized for exon capture using the Agilent SureSelect Human All Exon V6 kit and sequenced on the Illumina HiSeq *X* system according to the manufacturer's instructions. ANNOVAR was performed for functional annotation through various databases, including dbSNP, the 1000 Genomes Project, and HGMD. After filtering, the retained nonsynonymous SNVs were submitted to PolyPhen-2, SIFT, MutationTaster, LRT, M-CAP, and FATHMM for functional prediction. Sanger sequencing was then used to validate the mutation detected by WES in the proband and her parents. PCR amplification was performed with the ProFlex PCR System (Thermo Fisher Scientific, Waltham, MA, USA). DNA sequencing of PCR products was conducted on the ABI377 A DNA sequencer (Applied Biosystems). The primers used in the PCR analysis were as follows: F, 5′-AGTCATTGGTTTGGAATTAAAAATGCT-3′ and *R*, 5′-TGTGTTCTCTAAATAGCTGGAGACA-3'.

### 2.3. Plasmid Construction, Cell Culture, and Transient Transfection

Full-length *MMUT* cDNA was obtained from NCBI (https://www.ncbi.nlm.nih.gov). Plasmids carrying the wild-type and mutated *MMUT* cDNA were constructed by Wuhan Zhongmai Yingke Biotech Co., Ltd, China. HEK293T cells were grown on Dulbecco's modified Eagle's medium supplemented with 10% foetal bovine serum (Thermo Fisher Scientific, Waltham, MA, USA) and 1% penicillin/streptomycin. The cells were then transfected with plasmids encoding wild-type or mutant MCM using Lipofectamine 2000 (Invitrogen, Carlsbad, CA, USA). After 24–48 hours, the cells were harvested for further assays.

### 2.4. Real-Time PCR

Total RNA was extracted from HEK293 T cells with the TRIzol reagent (Invitrogen, Carlsbad, CA, USA) and was converted to cDNA using the RevertAid first-strand cDNA synthesis kit (Thermo Fisher Scientific, Waltham, MA, USA). Real-time PCR was performed using SYBR Premix Ex Taq II (TaKaRa) on the iCycler RT–PCR detection system (Bio–Rad Laboratories). The ΔΔCT method was used for data analysis [14]. Each assay was performed in triplicate for each sample. The GAPDH gene was used as an internal control. The primers for real-time PCR are given in Table 1.

### 2.5. Antibodies

The antibodies used in the western blotting were as follows: anti-Flag tag (1 : 500, 66008-2-Ig, Proteintech) and anti-GAPDH (1 : 5000, ab8245, Abcam). The anti-MCM mouse monoclonal antibody used for western blotting was kindly provided by Professor Ying Shen.

### 2.6. Western Blotting

Cell lysates were obtained by sonication in RIPA lysis buffer (Thermo Fisher Scientific, Waltham, MA, USA) and then centrifugated at 16 000 × g for 20 minutes at 4°C; the resultant supernatant was then used to investigate MCM expression levels. The protein supernatant was mixed with 5 × SDS loading buffer (Elabscience Biotechnology Co., Ltd., Wuhan, China) and heated to 100°C for 10 minutes. The denatured proteins were separated by 10% SDS-polyacrylamide gel electrophoresis and transferred to a polyvinylidene difluoride (PVDF) membrane (millipore) for immunoblot analysis.

### 2.7. Statistical Analysis

Statistical analyses were performed using SPSS version 17.0 software (IBM Company). Student's test and Fisher's exact test were used to compare the observed indices between experimental groups. *P* < 0.05 was considered significant.

## 3. Results

### 3.1. Case Presentation

**Figure 1:**
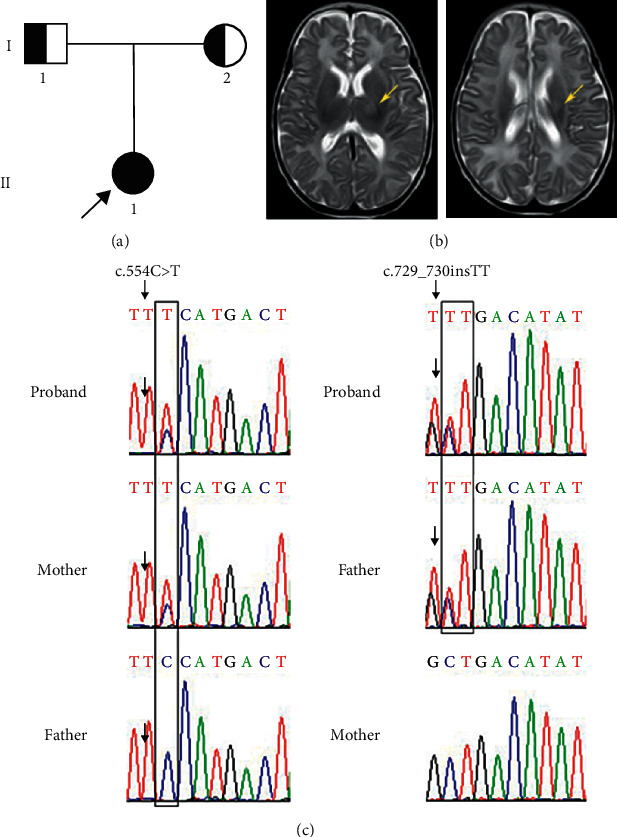
Clinical summary for the MMA family. (a) Family pedigree. An unrelated natural couple who gave birth to the affected child (black arrow denotes the proband). (b) The patient's axial brain MRI (yellow arrowheads show small patches of abnormal signals in the dorsal thalamic region of the left basal ganglia). (c) Sequence analysis of the human *MMUT* gene. A c.554C>T mutation of the *MMUT* gene was identified in the proband, and her mother was an asymptomatic heterozygous carrier. Arrows indicate the positions of the rare mutations. A c.729_730insTT mutation of the *MMUT* gene was identified in the proband, and her father was an asymptomatic heterozygous carrier. Arrows indicate the positions of the mutations.

A 3-year-old girl was born to nonconsanguineous parents (Figure 1(a)). Pregnancy, delivery, and the neonatal period were uneventful. The girl was born with a birth weight of 3050 g and was 50 cm in height. The child was presented at the hospital for the first time at the age of 2 months and 11 days; the main complaints were poor feeding and a reduced level of consciousness. She was admitted to the paediatric intensive care unit (PICU). Following a series of relevant examinations, various metabolic abnormalities were detected. For example, the blood ammonia concentration was >500 *μ*mol/L (normal range: 9–30 *μ*mol/L). Evaluations of metabolic disease using tandem mass spectrometry (MS/MS) revealed significantly elevated levels of propionyl carnitine and increases in the following ratios: Arg/Orn, Gly/Ala, Val/Phe, propionyl carnitine/free carnitine, propionyl carnitine/acetylcarnitine, and propionyl carnitine/hexadecanoylcarnitine. Analyses also revealed reduced free carnitine levels, Phe/Tyr ratios, and capryloyl carnitine/decanoic acid ratios. Gas chromatography/mass spectrometry (GS/MS) analysis showed extremely high concentrations of methylmalonic acid in urine. Magnetic resonance imaging (MRI) identified small patches of abnormal signals in the dorsal thalamic region of the left basal ganglia (Figure 1(b)). The patient was diagnosed with carnitine deficiency secondary to methylmalonic acidemia. Subsequently, the patient was required to take L-carnitine and mecobalamin supplements and adopt a low-protein diet. In subsequent evaluations, the patient showed significant improvement in her overall condition.

### 3.2. The Identification of Two Pathogenic Mutations in the MMUT Gene

**Figure 2:**
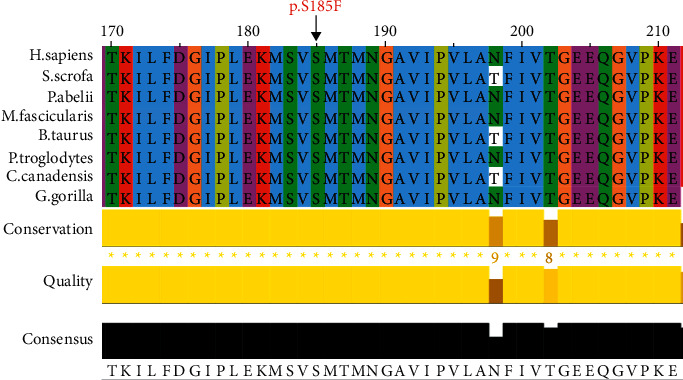
The conservation of *MMUT* variants. Multiple sequence alignment of the MCM protein for different species (black arrow denotes the position of the variant) (c.554C>T [p.S185F]).

To identify the underlying genetic cause of MMA in this family, we performed WES on all family members, including the father, mother, and the patient. The results of WES showed that the mother has a heterozygous c.554C>T (p. S185F) mutation in the *MMUT* gene, while the father carries a heterozygous variation of c.729–730insTT (p. D244Lfs^*∗*^39) in the same gene. Both the two heterozygous mutations were detected in the patient. Sanger sequencing further confirmed the compound heterozygous variations of c.554C>T and c.729–730insTT in *MMUT* in the patient (Figure 1(c)). The pathogenicity of the c.729–730insTT mutation in the *MMUT* gene remains certain. However, this rare variant of c.554C>T was classified as a variant uncertain of significance (VUS) according to the ACMG guidelines. It is also important to highlight that the site affected by this rare mutation shows high levels of conservation (100%) across many species (Figure 2).

To assess the potential effects of this rare missense mutation, we applied a range of bioinformatics software, including PolyPhen-2, SIFT, MutationTaster, LRT, M-CAP, and FATHMM. Analyses predicted that this variant is likely damaging (Table 2).

### 3.3. The c.554C>T Mutation Results in the Downregulated Expression of MMUT

**Figure 3:**
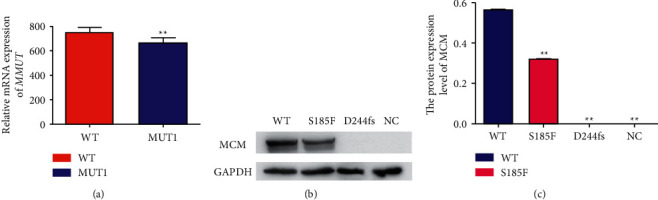
Effect of *MMUT* mutations on MMUT protein and mRNA levels in HEK293T cells. (a) The relative mRNA expression of *MMUT* was normalized to HEK293T cells transfected with the wild-type plasmid. The data collected from three independent experiments were subjected to statistical analysis (^*∗*^*P* < 0.05; ^*∗∗*^*P* < 0.01 vs. WT). (b-c) The relative protein expression was normalized to HEK293 T cells transfected with the wild-type plasmid. The data collected from three independent experiments were subjected to statistical analysis (^*∗*^*P* < 0.05; ^*∗∗*^*P* < 0.01 vs. WT). WT: wild type; MUT1: c.554C>T (p. S185F); MUT2: c.729–730insTT (p.D244Lfs^*∗*^39).

To verify the effects of the missense variant on the *MMUT* expression, we also constructed plasmids carrying the wild-type and mutated *MMUT* cDNA and transfected the plasmids into HEK 293T cells. We used quantitative real-time PCR to analyse the mRNA expression levels of *MMUT*. Compared with the cells transfected with the wild-type plasmid, the cells transfected with the mutant plasmid showed significant reductions in *MMUT* mRNA (Figure 3(a)). Furthermore, the expression levels of MMUT protein were then determined by the western blotting. Remarkably, a significant reduction in MMUT protein was observed in cells transfected with the mutant plasmid compared to the wild-type plasmid (Figures 3(b) and 3(c))).

## 4. Discussion

Defects in MCM or its coenzyme, cobalamin, lead to the accumulation of methylmalonic acid, which is characteristic of MMA [2–4]. MMA, first reported in 1967 [15], is a lethal, severe, and heterogeneous disorder of methylmalonate and cobalamin (cbl; vitamin B12) metabolism. The disease can be defined by MS/MS and GC/MS. Cases of MMA that are due to mutations in the *MMUT* gene usually lead to a severe phenotype and a very poor prognosis [16,17]. However, the known mutations of *MMUT* can only explain part of the affected individuals. In this study, we uncovered a rare mutation of c.554C>T in *MMUT* in a young girl suffering from MMA and first confirmed the negative effect of this mutation on the MMUT expression by functional experiment.

Given the poor prognosis of *MMUT*-related MMA, for example, varying degrees of psychoneurotic sequelae would lower the quality of patients' lives. The critical strategies to improve the outcomes of MMA patients are timely detection and treatment according to the current research situation. However, the pathogenicity of some new or rare mutations is uncertain, resulting in the limitation of the early diagnosis of MMA. Thus, more functional predictions of new or rare mutations in the *MMUT* gene need to be made, and more new pathogenic genes need to be discovered. Our study confirmed the harmfulness of the rare c.554C>T mutation in the *MMUT* gene, providing evidence for timely diagnosis and treatment. Meanwhile, our work further facilitated prenatal diagnosis and genetic counselling. In developing MMA prenatal diagnosis, preimplantation genetic diagnosis or screening (PGD/PGS) are the optimal effective prevention methods. It would be a prevailing trend to help those affected by MMA.

## 5. Conclusions

In summary, the rare c.554C>T mutation in *MMUT* identified in an MMA patient was suggested to be pathogenic. Our findings provide us with a greater understanding of the effects of *MMUT* variants and will facilitate the diagnosis and treatment of patients with MMA. Our work will play an important role in the prenatal genetic diagnosis of MMA. Raising sufficient awareness for mutation analysis of MMA can improve patient outcomes through timely treatment and benefit newborn screening strategies.

## Figures and Tables

**Table 1:** Primers used in the current study.

Primers	Forward	Reverse
MCM mRNA	5′ CTGGACCATCCGCCAGTATG 3′	5′ GCTAAAGTATAGGCCAGCTCCA 3′
Human GAPDH	5′ TGCACCACCAACTGCTTAGC3′	5′ GGCATGGACTGTGGTCATGAG 3′

**Table 2:** Analysis of the *MMUT* mutation in the MMA patient.

cDNA mutation	c.554C>T
Protein alteration	p.S185F
Mutation type	Missense allele frequency
gnomAD	NA
1000 Genomes	NA
SIFT_pred	Deleterious
Polyphen-2	Probably damaging
LRT_pred	Deleterious
MutationTaster_pred	Deleterious
M-CAP_pred	Deleterious
FATHMM_pred	Deleterious

NA: not available; a is the accession number for MUT GenBank:NM_000255.3.

## Data Availability

The datasets used and/or analysed during the current study are available from the corresponding author upon reasonable request.
